# Cellular Microenvironment in Human Pathologies

**DOI:** 10.1155/2013/946958

**Published:** 2013-11-07

**Authors:** Davide Vigetti, Martin Götte, Mauro S. G. Pavão, Achilleas D. Theocharis

**Affiliations:** ^1^Department of Surgical and Morphological Sciences, University of Insubria, 21100 Varese, Italy; ^2^Department of Gynecology and Obstetrics, Münster University Hospital, 48149 Münster, Germany; ^3^Instituto de Bioquímica Médica and Hospital Universitário Clementino Fraga Filho, Universidade Federal do Rio de Janeiro (UFRJ), 21941-590 Rio de Janeiro, RJ, Brazil; ^4^Department of Chemistry, University of Patras, 26500 Patras, Greece

It is well established that the cellular microenvironment dramatically influences cell behavior and is critical in many physiological functions, such as the differentiation niche of stem cells, or during development. Notably, it has pivotal roles in many pathologies. The surroundings of cells are constituted by several heterogeneous components forming a complex network of proteins and glycans. Although this extracellular matrix has been considered for decades as a mechanical scaffold where cells attach and reside, nowadays, it is known that such molecules modulate numerous cellular functions including survival, proliferation, migration, and differentiation. From a mechanistic point of view, the extracellular matrix components can interact with cells in several ways, including the modulation of soluble factor diffusion (i.e., growth factors, cytokines, morphogens, etc.), binding to their receptors, their subsequent activation, and regulation of signaling cascades. 

In addition to collagenous fibrillar structures, ECM is composed of proteoglycans, a heterogeneous group of secreted or membrane bound heavily glycosylated proteins. These sugars are represented by glycosaminoglycans that consist of repetitions of disaccharides formed mainly by an uronic acid with an amine sugar. These chains can be sulfated and modified in several ways leading to the formation of heparan sulfate, chondroitin sulfate, and dermatan sulfate. The only glycosaminoglycan without chemical modification is hyaluronan. Although simple in structure, hyaluronan is an information-rich molecule, and the functions of these glycosaminoglycans depend on the polysaccharide chain lengths. 

ECM turnover is fundamental in the physiological renewal of such components, and many degradative enzymes have also a role in pathology. Matrix metalloproteinases are probably the most well-known enzymes that catabolize many components of ECM including collagens and proteoglycans. More recently, other degradative enzymes specific for matrix polysaccharides have been shown to be critical in several physiological and pathological processes. Many growth factors and other mediators can bind to proteoglycans, and an altered matrix degradation can lead to an abnormal factor liberation. Further, hyaluronidases, producing low molecular weight hyaluronan, contribute to inflammation. 

An increasing body of literature suggests that an altered cellular microenvironment is mechanistically involved in a variety of human pathologies. Interestingly, several extracellular components influence neoplastic diseases and cardiovascular, pulmonary, and renal pathologies that, together, represent the major causes of death in industrialized countries.

In this special issue comprising review articles and original research papers, all the above topics are deeply discussed by experts in the field.

Two works are focused on the skeletal system; M. Maldonado and J. Nam discuss the changing of ECM components in the presence of inflammation during osteoarthritis whereas S. A. Syggelos and collaborators review the effects of matrix metalloproteinases in periprosthetic loosening and osteolysis.

Different aspects of renal disorders are presented in the works of J. A. Lepedda and coauthors, who describe the use of bikunin as a marker of Fabry's disease, and S. P. Srivastava and coresearchers, who provide an overview on the role of microRNA in fibrosis and diabetic nephrology.

The effect of biomechanical forces in the tissue microenvironment on the regulation of fibrosis is deeply reviewed by W. Carver and E. C. Goldsmith.

ECM effects on cardiac primitive cell properties and their role in normal and pathological human heart are presented by C. Castaldo and collaborators.

The effects of low-density lipoprotein receptor-related protein-1 (LRP-1) on matrix remodeling are deeply reviewed by N. Etique and coworkers.

S. Plantman reviews the role of ECM in axonal and neuronal regeneration. 

Many papers are focused on cancer and metastasis and report the effect of several ECM components in different types of neoplasia.

The role of hyaluronan and its receptors CD44 and RHAMM on fibrosarcoma progression is presented by D. Nikitovic and coresearchers.

The effects of epidermal growth factor receptor on digestive tract cancers are discussed by T. Sasaki and coauthors.

Two works focused on breast cancer; A. M. Gomes and collaborators show the effects of heparanase and heparan sulfate on tumor progression, whereas D. Lymperatou and coauthors present the effects of fulvestrant and tamoxifen on cell migration.

S. Mizumoto and coresearchers present the effect of N-acetylgalactosamine 4-sulfate 6-O-sulfotransferase in pulmonary metastasis.

The role of prostate stem cells in development of benign prostate hyperplasia or prostate cancer is reviewed by A. Prajapati and collaborators.

Syndecan-4 expression and correlation with metastatic potential in testicular germ cell tumors are presented by V. T. Labropoulou and coworkers.

Overall, the papers presented in this special issue not only underscore the importance of the cellular microenvironment for a variety of physiological and pathophysiological processes with considerable relevance for human disease, but also provide insight into the molecular diversity of regulatory interactions exerted by the microenvironment on a wide range of cell types. A deeper understanding of the functional properties of the cellular microenvironment thus emerges as an important prerequisite for the development of more efficient therapeutic approaches in tissue repair and regeneration and diseases ranging from chronic and acute inflammation to cancer. 

*Davide Vigetti* *Martin Götte* *Mauro S. G. Pavão* *Achilleas D. Theocharis*

